# The Molecular Pathway of Argon-Mediated Neuroprotection

**DOI:** 10.3390/ijms17111816

**Published:** 2016-10-31

**Authors:** Felix Ulbrich, Ulrich Goebel

**Affiliations:** Medical Center, Department of Anesthesiology and Critical Care, Faculty of Medicine, University of Freiburg, Hugstetter Strasse 55, 79106 Freiburg im Breisgau, Germany; felix.ulbrich@uniklinik-freiburg.de

**Keywords:** argon, neuroprotection, cytoprotection, molecular pathway, mitogen-activated protein kinases, heat shock proteins, cytokines, transcription factor, toll-like receptors

## Abstract

The noble gas argon has attracted increasing attention in recent years, especially because of its neuroprotective properties. In a variety of models, ranging from oxygen-glucose deprivation in cell culture to complex models of mid-cerebral artery occlusion, subarachnoid hemorrhage or retinal ischemia-reperfusion injury in animals, argon administration after individual injury demonstrated favorable effects, particularly increased cell survival and even improved neuronal function. As an inert molecule, argon did not show signs of adverse effects in the in vitro and in vivo model used, while being comparably cheap and easy to apply. However, the molecular mechanism by which argon is able to exert its protective and beneficial characteristics remains unclear. Although there are many pieces missing to complete the signaling pathway throughout the cell, it is the aim of this review to summarize the known parts of the molecular pathways and to combine them to provide a clear insight into the cellular pathway, starting with the receptors that may be involved in mediating argons effects and ending with the translational response.

## 1. Introduction

Argon is the third noble gas, part of the eighth main group in the periodic table of elements with an atomic number of 18. It is a one-atom, odorless and extremely inert gaseous molecule, condensing only at temperatures below 87.15 K. Among the noble gases, argon is the most common in the atmosphere with approximately 9340 ppm [1].

After Henry Cavendish realized in 1784 that air is composed of a third (and so far unknown) fraction beside oxygen and nitrogen [2], it was only in 1894 that Lord Rayleigh and Sir William Ramsay confirmed his results and—for the first time—synthesized argon through fractionated distilling of liquefied air [3]. One of argon’s physico-chemical characteristics is a full electron valence shell, which in turn prevents any covalent bindings with other substances. Hence, the newly discovered molecule possessed only slow chemical properties if any, and it was therefore named argon (greek: “αργόν” meaning lazy).

Although called “inert”, noble gases may exert certain biological effects, especially interacting with larger proteins, protein cavities or even receptors [4]. Because of its atomic structure, argon is able to bind to certain receptors recruiting stabilization energy such as charge-induced dipoles or van-der-Waals forces [5,6], thus inducing biologically relevant actions such as anesthesia or neuroprotection. The anesthetic effects were first described by Behnke, analyzing the effects of argon as an additional gas used for deep sea diving. Argon was inhaled at 69% in four divers, who were pressurized under water to approximately 130 feet depth. The authors conclude: “*(A) The narcotic effect of argon is greater than that of nitrogen at high pressures of 4 to 10 atmospheres, corresponding to depths of 100 to 300 feet. (B) At a pressure of 1 atmosphere no difference could be detected between argon, nitrogen, or helium with respect to respiratory resistance or psychologic effects*” [7]. As a non-polar molecule, argon may easily diffuse into tissues like the deeper brain compartments, there interacting with mostly amphiphilic proteins even within receptor cavities [8].

In recent years and beside the well-established gaso-transmitters carbon monoxide, hydrogen sulfide and nitric oxide [9], argon has attracted some attention as a promising new player in the group of potentially neuro- and organoprotective substances. In contrast to the other substances, unwanted or even detrimental side effects have not been detected so far.

A variety of different substances have been tested pre-clinically regarding their neuroprotective potential in the last years [10]. However, clinical evaluation of multiple neuroprotective strategies and substances either showed weak effects only, or failed to prove relevance in clinical practice at all. Although there is no such thing as a “magic bullet”, it is still most tempting to speculate that argon may provide powerful neuroprotective effects not only in experimental setting but in clinical settings as well.

While argon has been demonstrated to prolong survival or increase neuronal function in certain experimental settings of neurological injuries, the molecular basis of these effects remains the subject of ongoing research. This review will discuss the molecular findings of argon-mediated beneficial and protective effects in various models of neuronal injuries, including receptor interactions, anti-inflammatory and anti-apoptotic pathways as well dose and time dependency after argon administration.

## 2. Neuroprotection

The concept of “neuroprotection” in our current understanding was first introduced in 1987 by Gill et al. using Dizocilpine, an antagonist of the *N*-Methyl-d-aspartate (NMDA) receptor, in a hippocampal ischemia and reperfusion model, showing potent effects [11]. Neuroprotective drugs or interventions aim to prevent or slow disease progression including secondary injuries, by interrupting neuronal apoptosis and thus the loss of neurons and their functions [12]. Although there is a huge variety in central nervous system-associated injuries or symptoms, the mechanisms of neurodegeneration are quite similar. Among possible mechanisms considered essential in the contribution or the maintenance of neuroprotection, the most promising ones to serve as targets for therapeutic interventions are inflammation, apoptosis, mitochondrial dysfunction, protein aggregation and excitotoxicity [13].

Apart from some physiological studies regarding the anesthetic potency of argon [14,15] and long-term analysis of work performance under argon-saturated atmosphere [16], it was up to Soldatov and co-workers to first describe argon’s neuroprotective properties in 1998. After hypoxic brain injury, rats were treated with argon inhalation, applying concentrations ranging from 25% to 77%. Thereafter these rats showed a significantly reduced area of brain damage and improved survival compared to control animals [17]. In the last two decades several scientists have confirmed argon’s beneficial effect in vitro and in vivo [18].

## 3. Argon as a Neuroprotective Gaseous Molecule in Various Models of Injury

The effects of argon were tested in different in vitro and in vivo models. Regarding cell-culture based research, injury of neuronal cells by deprivation of glucose and oxygen is a frequently used model, investigating neuroprotective properties of gaseous molecules.

In 2009, Jawad et al. [19] treated neuronal cell cultures from fetal mice cortices with oxygen-glucose-deprivation (OGD) for 90 min in an atmosphere of 95% N_2_ and 5% CO_2_ or 75% argon, 20% N_2_ and 5% CO_2_. Following incubation in an argon-enriched environment for 24 h (75% argon, 20% O_2_, 5% CO_2_) neuronal cells showed an increased viability compared to the control group, receiving OGD without argon treatment.

Loetscher et al. [20] exposed slices of mice hippocampal tissue to OGD for 30 min (95% N_2_, 5% CO_2_ or 25%–74% argon, 5% CO_2_, rest N_2_). Then, slices were incubated with argon for 72 h in concentrations ranging from 25% to 74% (21% oxygen, 5% CO_2_, argon, remainder N_2_) immediately after injury or within a time delay of 3 h. Argon reduced neuronal injury showing the strongest effect at a concentration of 75%. Even with a delay of three hours, argon (50%) still reduced OGD mediated neuronal injury significantly. In this study Loetscher et al. [20] demonstrated for the first time a dose- and time-dependent effect of argon in vitro.

David et al. [21] confirmed argon’s neuroprotective effect in vitro, at least in part. Coronal brain slices were injured by OGD for 20 min in glucose-free solution saturated with 100% N_2_. Immediately after this OGD injury, slices were incubated with medical air or argon in three different concentrations (25%–75% argon, O_2_ 25%, remainder N_2_) for 1 up to 3 h. In this study, lactate dehydrogenase (LDH) as a marker of brain damage was reduced maximally after exposition with argon 50% (50% argon, 25% O_2_, 25% N_2_). Interestingly, argon’s neuroprotective effect intensified with increasing exposition time and reached a maximum at 3 h.

Zhao and co-workers [22] injured cortical cells by OGD (95% N_2_ and 5% CO_2_ or 75% argon, 20% N_2_ and 5% CO_2_ for 90 min). Afterwards, argon was administered for 2 h (70% argon, 25% O_2_, 5% CO_2_) and reduced OGD induced cell death compared to cells not exposed to argon.

Investigating neuroprotection in rodents, models of cerebral ischemia like the transient middle cerebral artery occlusion model (tMCAO) were commonly used.

In 2011, Ryang et al. [23] performed tMCAO for 120 min in rats. With a delay of 1 h after retraction of the filament from the central artery, animals received either argon for 1 h (50% argon, 50% O_2_) or nitrogen (50% N_2_, 50% O_2_). In argon-treated animals, a decreased infarct size in the cortex area and basal ganglia was found, but interestingly not in the hippocampal area. However, overall infarct size was significantly reduced compared to nitrogen treatment. Moreover, neurological outcome based on the neurologic deficit score was improved after exposure to argon.

David et al. [21], too, induced neuronal cell death using the tMCAO (for a duration of 60 min) in rats. With a delay of 1 h after reperfusion, animals received either argon for 3 h (50% argon, 25% O_2_, 25% N_2_) or medical air. Animals exposed to argon showed a decreased cortical volume of brain damage. However—and in contrast to all other studies—subcortical brain damage was increased after argon treatment and neurological outcome was worsened compared to SHAM-treated rats.

In 2014, Fahlenkamp et al. [24] performed 2 h of tMCAO in rats. After one hour of tMCAO, animals received argon (50% argon, 50% O_2_) or placebo (50% N_2_, 50% O_2_) for the next tMCAO hour via face mask. In this study, after argon exposition no significant differences in vital neurons, microglia and astrocytes were found in healthy tissue, ischemic area or penumbra compared to the control group.

Simulating neonatal asphyxia, Zhuang et al. [25] performed right common carotid artery ligation and exposed neonatal rats to hypoxic conditions for 90 or 120 min (92% N_2_, O_2_ 8%). Subsequently, rats were exposed to air (control group) or argon (70% argon, 21% O_2_, remainder N_2_) for another 90 min. Compared to the control animals they found an increase of hippocampal cell viability within the argon group.

Zhao et al. [22] confirmed these findings in 2016. After unilateral common carotid artery ligation, rat pups were exposed to a hypoxic atmosphere (O_2_ 8%) for 90 min. Thereafter, animals were exposed to argon (70% argon, 30% O_2_). In this study, argon decreased neuronal cell death and reduced brain infarction significantly.

In a model of cardiac arrest, where neurological outcome was secondary to the injury, Brücken et al. [26] confirmed argon’s neuroprotective effect. After induction of cardiac arrest and one hour after successful cardiopulmonary resuscitation, Sprague-Dawley rats received either 70% argon or 70% nitrogen (added to O_2_ 30%) for 1 h. After argon treatment, neuronal damage was significantly decreased in neocortex and hippocampal (Cornu Ammonis = CA) CA3/4 sections. Neurologic outcome, measured with the neurological deficit score (NDS) and in an open field test, was improved. Even with a 3 h delay after successful resuscitation, argon treatment still reduced neuronal damage significantly. Compared to nitrogen ventilation (70% N_2_, 30% O_2_), even lower concentrations of argon mediated neuroprotection as well.

Ischemia and reperfusion (IRI) may be evaluated by strict standardization in the model of retinal IRI, which is frequently used by Ulbrich et al. [27]. Rats receive retrograde labeling of retinal ganglion cells using fluorogold. One week later, the left eyes are cannulated using a 30-gauge needle, increasing pressure to 120 mmHg for one hour. After release of this pressure and reperfusion, the animals received argon either directly or with a time delay of 1.5 or 3 h in concentrations ranging from 25%–75% argon (O_2_ 21%, rest N_2_). The surviving retinal ganglion cells were counted 7 days later, showing a time- and dose-dependent protection of these neuronal cells through argon treatment [27].

Höllig et al. [28] recently published the results of a new model, inducing brain injury by subarachnoid hemorrhage in rats. One hour after an intracranial endovascular perforation procedure, animals received argon 50% (50% O_2_). Compared with the control group argon, reduced the risk with respect to premature death to 20.6%. The quantity of NeuN-positive nuclei—as a biomarker of vital neurons—was significantly elevated in argon treated animals (dentate gyrus, not CA1 or CA3/4) 24 h after injury.

These promising results of neuroprotection in different models all raise two questions: How does argon exert these effects, and what is the molecular mechanism?

## 4. Receptor Mediated Neuroprotection

Is it possible that a gaseous molecule thought to be inert and chemically non-reactive can interact with extracellular located receptors? At least, the noble-gas xenon exerts its well-known narcotic effect by antagonizing the NMDA-receptors [29] and in turn provides neuroprotective effects at low concentrations [30,31,32,33,34]. Therefore, irrespective of the NMDA-R, a variety of receptors have been examined, trying to prove a causal link to argon-mediated neuroprotection.

In 2003, Abraini et al. [35] were the first to analyze the effect of argon in relation to specific receptors. At high pressure, argon exerts relevant anesthetic potency. Abraini found that pretreatment with gabazine, 2-hydroxysaclofen, and flumazenil required increased pressure to induce anesthesia with argon, compared to control rats. They therefore concluded that the gamma-Aminobutyric acid (GABA)_A_ receptor is part of argon mediated anesthetic effects at high pressure. Moreover, it seemed reasonable that noble gas effects are comparable to those of generally used volatile anesthetic agents, but the authors could not prove that argon provides any protective effects via the GABA_A_ receptor.

Ten years later, Harris et al. [36] examined argon’s neuroprotective effect on traumatic injury of hippocampal brain slices with special attention to the involvement of NMDA-receptors and the two-pore domain K^+^-channels (TREK-1). After ex vivo injury was set with a stylet and standardized force (3.5 µJ), brain slices (CA1 region) were immediately incubated within an argon gas mixture (0.5 atm argon, 0.2 atm O_2_, 0.05 atm CO_2_, 0.75 atm N_2_) for 30 min and up to 72 h. Harris showed that argon treatment mediated neuroprotection and reduced traumatic injury, determined by visualization of PI fluorescence intensity, at all timepoints measured (i.e., 24, 48 and 72 h) compared to the injured slices without argon treatment. However, in contrast to identical experiments using xenon, administration of glycine did not attenuate argon’s effect, therefore indicating a mechanism independent of NMDA-receptor inhibition. Furthermore, electrophysiological tests demonstrated that argon had no influence on NMDA receptor currents. In addition, argon did not affect halothane-induced currents via two-pore domain K^+^-channels (TREK-1) in transfected HEK-293 cells. Although a clear mechanism could not be demonstrated in this study, there is good evidence that NMDA- and TREK-1 receptors are not part of argon-mediated neuroprotection.

In the next attempt to find the receptor accountable for argon-mediated neuroprotection, Brücken et al. [37] investigated argon’s effect in a cardiac arrest model analyzing mitochondrial K_ATP_-channels. A bolus of 9 mg/kg 5-Hydroxydecaonate (5-HD) was administered 50 min after return of spontaneous circulation (ROSC) and 10 min before starting argon ventilation (40% or 70% argon, oxygen respectively) blocking K_ATP_-channels. While argon treatment showed the expected and dose-dependent moderate to strong neuroprotective effects in the brain (mainly in the neocortex and in the hippocampal area CA3/4), a significantly improved neurological deficit score throughout the first seven days after resuscitation and increased mobility in the open field test, the authors found that a K_ATP_-channel blockade (via 5-HD) did not change argon’s effects. They concluded that argon does not open the K_ATP_-channel and its neuroprotective effects are thus mediated differently.

Ulbrich et al. [38] performed investigations concerning argon’s mechanism in vivo and in vitro. In a neuroblastoma cell line, rotenone was used as an inhibitor of the mitochondrial respiratory chain, inducing apoptosis. Argon exposition, applying different concentrations for 2 or for 4 h (25%–75% argon, 21% O_2_, remainder CO_2_/N_2_), mediated cell protection and exhibited the best effects at 75%. Argon significantly reduced FACS-associated antibody binding to surface expression of toll-like receptors (TLR) 2 and 4. These results were confirmed by immunohistology. This was interpreted as a clear hint of an argon-receptor interaction. In contrast TLR3, TLR6, TLR8 and TLR9 were not affected. Intracellular blocking of TLR4 abolished argon’s neuroprotective effect partly, while inhibition of both, TLR2 and TLR4 completely annulled argon’s effects. These findings were then translated and confirmed in vivo: In a standardized model of retinal ischemia-reperfusion injury argon inhalation for 2 h (75% argon, 21% O_2_, remainder N_2_) protected retinal ganglion cells und decreased retinal TLR2 and 4 expression. Inhibition of TLR2 and TLR4 signaling attenuated argon’s neuroprotective effects completely [39]. The authors concluded that TLRs are responsible for argon-mediated effects.

It is most likely that not only toll-like receptors interact with argon to mediate its specific effects. Ongoing research is not restricted to neuronal injury, but also include renal and pulmonary impairment, as the mechanism should be similar [40].

## 5. Intracellular Pathways Displaying Argon-Mediated Neuroprotection

The first manuscript describing a possible molecular mechanism of argon’s neuroprotective effect in vitro was published by Fahlenkamp et al. in 2012 [41]. The authors subjected primary neuronal and astrocyte cultured cells of mice bulbs as well as BV-2 microglia cells to lipopolysaccharide (LPS). They exposed cells to argon (50% argon, 24% N_2_, 21% O_2_ and 5% CO_2_) for 15, 30, 60 and 120 min, analyzing the phosphorylation of extracellular signal-regulated kinase (ERK)-1/2. Activation of ERK1/2 may play a critical role in neuronal apoptosis triggered by cellular damage [42] and its activation is crucial for its later role in promoting cell death or cell survival [43]. ERK1/2 phosphorylation was seen with a really quick onset after argon exposition. Within 30 min, ERK1/2 phosphorylation could be detected, while it was only short lasting. Argon effects regarding ERK phosphorylation could be inhibited using the MEK-inhibitor U0126. In contrast to the known effects of xenon [44], argon did not alter LPS-mediated cytokine profile. Recently, Zhao et al. [22] were able to confirm the role of ERK1/2 in their 90 min OGD model in cortical neuronal cell culture of rat foetus. In addition, ERK inhibition using FR180204 directly suspended argon’s effects regarding ERK phosphorylation with subsequent increase in apoptosis in a neuroblastoma cell line, analyzed by Ulbrich et al. [38]. In an in vivo model of retinal IRI, Ulbrich et al. [45] were able to prove these effects in rats; an inhibition of ERK1/2 administering PD98059 intravenously significantly reduced argon’s neuroprotective effects. The authors were able to show that these ERK1/2-mediated effects are responsible for an increase in caspase-3 cleavage and subsequent in apoptosis. Other mitogen-activated protein kinase, like p38 or the c-Jun N-terminal kinase (JNK) are not differentially phosphorylated due to argon treatment. This is interesting, since other volatile agents (xenon, volatile anesthetics or carbon monoxide) generally activate more than one mitogen-activated protein kinase to promote their intracellular signaling [46].

Zhuang et al. [25] confirmed argon’s possible beneficial and anti-apoptotic effect. In their model of neonatal asphyxia the authors found that argon treatment did not show any effects regarding the phosphorylation of pro-apoptotic proteins BAX and BCL-XL, but a significant increase of the anti-apoptotic acting protein BCL-2. Zhao et al. [47] demonstrated in vitro and in vivo that argon administration after neuronal injury may upregulate BCL-2 expression, while caspase-3 expression was cut back to baseline levels in the cortex and the hippocampal area of MCAO injured rats. Ulbrich et al. [27] confirmed these findings of argon’s anti-apoptotic properties in a different in vivo model of retinal ischemia and reperfusion injury (IRI). While BLC-2 mRNA expression was shown to be time- and dose-dependently suppressed in the context of argon treatment, mRNA expression of BAX was only suppressed due to high concentrations of argon (i.e., 75%). Furthermore, the authors demonstrated that IRI-induced caspase-3 cleavage was suppressed by argon 75%, if administered immediately after injury. Extended FACS analysis by Zhao et al. and Ulbrich et al. confirmed the anti-apoptotic properties of argon, as well as results, proving a reduction in reactive oxygen species (ROS) formation and normalization of mitochondrial membrane potential after argon treatment [38,39,47].

Argon mediated reduction in caspase-3 activity seems to be dependent on TLR2 and TLR4 activation. Ulbrich et al. [39] were able to demonstrate that the inhibition of TLR2, TLR4 or both significantly inhibited argon-mediated protective effects in vitro. Of note, argon’s effects were accompanied by a suppression of interleukin-1-receptor-associated kinase 4 (IRAK4), but no alteration was found in myeloid differentiation primary response gene 88 (MyD88), while both proteins are thought to be a crucial part of TLR downstream signaling. These results suggest that argon blocks TLRs, furthermore stopping intracellular downstream signaling.

Toll-like receptors, IRAK and especially ERK1/2 are to activate transcription factors, such as nuclear factor kappa-light-chain-enhancer of activated B cells (NF-κB), signal transducer and activator of transcription (STAT3) or nuclear factor (erythroid-derived 2)-like 2 (Nrf2) [48]. Zhao et al. [22] were able to demonstrate a significant increase in Nrf2 due to argon treatment in vitro and in vivo. This effect was accompanied by increased expression of p-mTOR and Nrf2 down-stream effectors nicotinamide adenine dinucleotide phosphate (NADPH) dehydrogenase, NADPH quinone dehydrogenase-1 (NQO1) and superoxide dismutase 1 (SOD1). Ulbrich et al. [27] demonstrated a sustained phosphorylation of NF-κB compared to positive controls due to high-dose argon treatment in vivo, showing a time dependency after injury. They concluded that NF-κB, as a potential modulator of apoptosis, may be a downstream target, exerting distinct effects on neuronal cells. These findings were later on supported by Zhao et al. [47] in an in vivo model of neonatal hypoxia and brain injury. This argon-mediated suppression of NF-κB and STAT3 signaling was counteracted by TLR inhibition in vitro, rendering these possible pathways. Interestingly, no effects were detectable for signal transducer and activator of transcription (STAT) 5 and cAMP responsive element binding protein (CREB), although these two transcription factors are known to play a role in neuroregeneration [39]. It is more than likely that even more transcription factors are involved in the pathway from surface receptor to intra- and extracellular effect-sides of argon-mediated neuroprotection, which remain part of future investigation.

The mitogen-activated protein kinases as well as transcription factors like NF-κB, STAT or Nrf2 are known to be part of the heat shock response [49]. Ulbrich et al. [45] examined argon’s influence regarding the expression of heat shock proteins in vivo. After retinal IRI in Sprague-Dawley rats, the 70 and 90 kDa heat shock proteins as well as the heme-oxygenase-1 (HO-1, HSP-32) were markedly increased. Following argon inhalation (75%, O_2_ 21%, remainder N_2_), this heat shock response was attenuated significantly. Inhibition of ERK1/2 increased HO-1 expression again, but not HSP-70 or HSP-90. It is tempting to speculate that HO-1 may be the central and common part in different settings of protection. Zhao et al. [47] confirmed the findings with respect to HO-1 in their in vitro OGD and hypothermia model as well as in vivo argon-mediated neuroprotective effects through HO-1 induction. These effects were prominent in both, the cortex and the hippocampal area, while the brain infarct volume was reduced. Although the exact pathway still includes questions and unknown areas, PI3K/p-Akt and GSK-3β may be involved in the HO-1 dependent regulation of argon-mediated neuroprotection.

After induction of subarachnoid hemorrhage (SAH) in rats, Höllig et al. [28] performed Western blot analysis for HO-1 and HIF-1α protein of hippocampal tissue at different time points. The results are difficult to interpret; while argon treatment reduced HO-1 protein expression at 6 h after SAH, HO-1 was ultimately induced at 24 h. Furthermore, HIF-1α protein expression was significantly increased only after 24 h due to argon inhalation, while both proteins failed to show any elevated expressions at 72 h post injury. Heme oxygenase-1 may be induced via HIF-1α, but this is not the usual pathway; Nrf2 phosphorylation or heat shock factor-1 (HSF-1) trimerisation and subsequent translocation are predominantly considered the transcription factors of activation [50]. Moreover, both proteins are stress proteins which react fast to guard and protect cells ahead of further harmful events. On the other hand, these result suggest that argon is still able to activate cellular defense mechanisms and change cells fate in the penumbra region even 24 h after injury, making argon an even more promising therapeutic candidate.

To date, the results regarding argon-mediated anti-inflammatory signaling (especially argon mediated cytokine response) are still controversial. Fahlenkamp et al. [41] investigated in vitro which effects argon 50% (applied for 4 h) may exert in the context of an LPS induced inflammation. Analyzing three cytokines (i.e., IL-1β, TNF-α and IL-6), the authors found that only IL-1β was significantly reduced due to argon treatment, but not TNF-α and IL-6. In contrast, Zhao et al. reported that argon reduced the expression of the inflammatory cytokines TNF-α and IL-6 after hypoxic-ischemic brain injury in cortical tissue in rats [22].

In a complex study, Fahlenkamp et al. [24] extensively analyzed 16 different genes of possible interest, which could be altered in their individual expression due to the treatment with argon; the model used to analyze argon’s effects was a 2 h tMCAO model with inhalative argon 50% treatment for one hour afterwards. The authors compared tissue of the penumbra after tMCAO with the respective side of the contralateral hemisphere 24 h after injury. The contralateral and comparable side of the hemisphere was not affected by any one protein tested due to argon treatment. The expression of inflammatory genes showed various results: CD3 (part of the T-cell receptor and therefor responsible for a variety of inflammatory stimuli) was suppressed due to argon inhalation, compared to control animals, while CD11b expression (a macrophage marker) was not responsive. In contrast, IL-1β, IL-6 and iNOS were expressed at significantly higher levels after argon treatment, while TNF-α expression remained unchanged. Analyzing different growth factors, the authors found that the nerve-growth factor (NGF) and the transforming-growth factor (TGF)-β were increased in their expression after tMCAO and argon treatment, while the vascular endothelial growth factor (VEGF)-α and the insulin-like growth factor (IGF-1) did not show any alterations. No changes in the argon-mediated expression pattern were observed for the hypoxia inducible factor-1α (HIF-1α), matrix metalloproteinase-9 (MMP9), C-type natriuretic peptide (CNP) or glial fibrillary acidic protein (GFAP).

Ulbrich et al. [39] investigated argon’s effect on TLR-dependent inflammatory cytokines in human neuroblastoma cells (SY5Y) after rotenone treatment. While rotenone was able to induce several inflammatory cytokines like TNF-α, IL-1β, IL-6, IL-8 und IL-10, only IL-8 was affected by argon treatment. Interestingly, argon did not increase any of the named cytokines, but significantly decreased IL-8 mRNA and protein expression. Subsequently, these results were transferred in vivo. Retinal ischemia-reperfusion injury increased IL-8 protein expression in the ganglion cell layer. While argon treatment significantly reduced IL-8 expression, inhibition of TLR2 and TLR4 abolished argon’s effect. Interestingly, retinal IRI led to an increase in serum IL-8 levels, which responded the same way to both argon treatment and inhibition of TLR2 and TLR4.

In 2014, Ulbrich et al. [27] already reported a sustained decrease in the white blood cell count of rats treated with argon inhalation (75%) after retinal IRI, compared to control animals, which showed expected high levels of white blood cells in response to retinal IRI 24 h after injury. These argon effects were only observed if animals were treated immediately. Any time delay in treatment canceled this effect. Systemic effects of neuro-inflammation, especially a decrease in IL-8, are commonly observed in neurodegenerative diseases like Parkinson’s disease, thus opening new treatment opportunities for argon in different neurological disease [51].

A table summarizing the proposed mechanisms of argon mediated neuroprotection is found in Table 1.

## 6. Conclusions

Although the scientific community has to deal with a variety of different models in which protective effects of argon have been investigated (predominantly tMCAO, OGD, retinal-IRI and subarachnoid hemorrhage), the neuroprotective properties of argon are widely confirmed by different authors. The proposed mechanism for argon mediated neuroprotective effects includes TLR2 and TLR4 signaling, while the NMDA, TREK-1, K_ATP_ and GABA_A_ receptors do not interact with argon under normobaric pressure. Although there is still some controversy regarding the involvement of other cell-surface based receptors, the intracellular molecular pathway of argon’s effects shows unambiguous assignment and seems well accepted. Among the clearly proven steps, ERK1/2 phosphorylation in the context of HO-1 activation is crucial, mediating anti-apoptotic signaling via BAX, BCL-2 regulation and caspase-3 activity.

While there is a certain consent about argon’s anti-apoptotic effects, the results regarding the (anti)-inflammatory effects need to be analyzed in future studies.

In general, the mechanism of argon-mediated neuroprotection is still not understood completely, but new evidence sheds some light on the molecular pathway. Since the argon molecule is of substantial size, it seems reasonable that there needs to be a receptor-associated pathway, signaling argon’s effects inside neuronal cells. While extensive experiments were carried out, today we can be sure that argon mediates narcotic effects either via the NMDA or the GABA_A_ receptors. But these effect were only seen under hyperbaric conditions, and the mechanism was more a conclusion than an experimental analysis. Under normobaric conditions, neither receptor (NMDA and GABA_A_) plays a role in argon-mediated neuroprotection. Moreover, the TREK-1 and the K_ATP_ channel, which have been analyzed in vitro as well as in vivo, are not part of the argon-mediated effects. Ulbrich et al. [38] analyzed six toll-like receptors, because the known intracellular pathway (including the obviously necessary phosphorylation of ERK1/2, as well as the suppression of heat-shock proteins and the balance of mitochondrial proteins due to argon administration) points to one common structure, i.e., the toll-like receptors. Among these six TLRs analyzed, only two of them showed specific extracellular modification and intracellular blocking of TLRs abandoned argon’s effects. Of note, argon mediates its effects via TLR2 and TLR4, while TLR4 seems to have more effect than TLR2. This potential pathway was confirmed in a series of in vivo experiments, where argon had no neuroprotective effects anymore, after the intracellular inhibition of TLR2 and TLR4; argon’s extracellular binding to the surface receptor was not altered, but intracellular signaling was stopped.

The difference in protein structure concerning the affinity of argon to TLRs and not any other receptor like TREK-1 or other receptors has to be investigated in the future. X-ray analysis of the solid-state structure of the receptor molecules may help to solve this question of why argon is attracted to TLRs, but not to other receptors. Toll-like receptors are widely known in mediating inflammatory signals (especially in sepsis) and stimulation is usually done by substances like LPS. By contrast, apoptotic signaling via TLRs is rather uncommon in the current literature.

**Figure 1:**
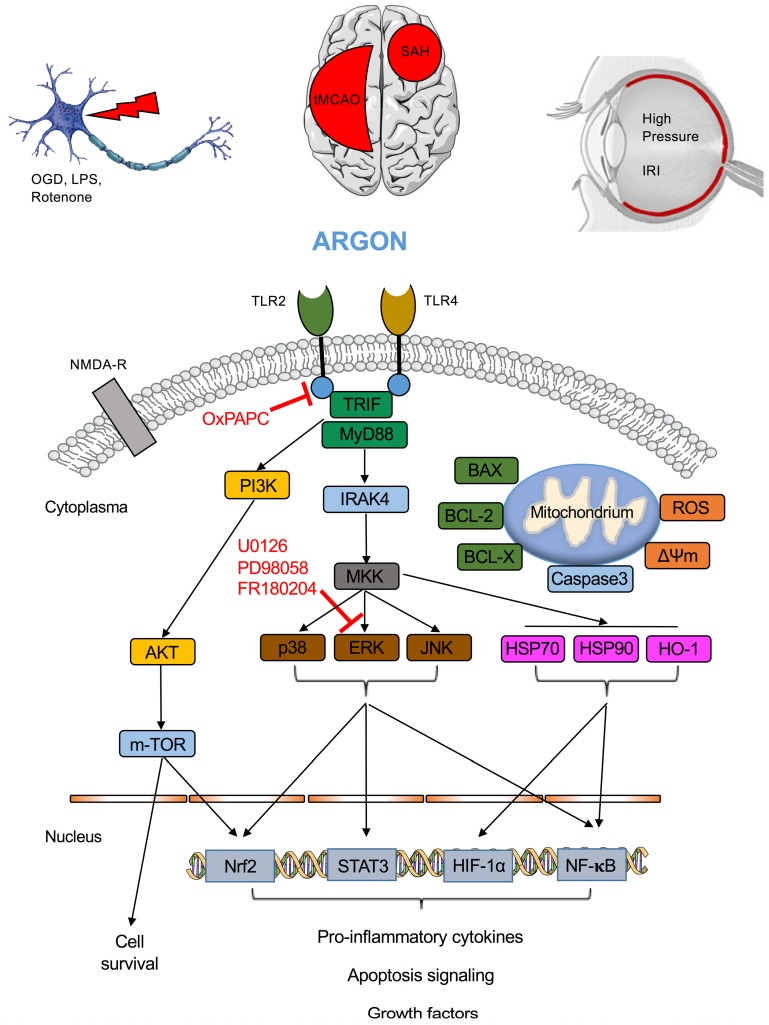
Argon’s molecular mechanism. Argon is able to specifically activated toll-like receptors 2 and 4, mediating intracellular signaling via IRAK4 and ERK1/2 (and to some amount p38) and inhibiting the heat shock response (HSP-70, -90 and HO-1) or activating the PI3K/mTOR pathway. Mitochrondrial signaling includes Bcl-2, Bcl-X and BAX expression, while ROS is reduced and mitochondrial membrane potential remains constant. These intracellular proteins lead to a differential activation or suppression of transcription factors, thus inducing or inhibiting effector genes and proteins (e.g., IL-8). OGD = oxygen-glucose deprivation, LPS = lipopolysaccharide, tMCAO = transient mid cerebral artery occlusion, IRI = ischemia and reperfusion injury, SAH = subarachnoid hemorrhage, NMDA-R = *N*-methyl d-aspartate receptor, TLR = toll-like receptor, TRIF = TIR-domain-containing adapter-inducing interferon-β, MyD88 = myeloid differentiation primary response gene 88, IRAK4 = interleukin-1 receptor-associated kinase 4, PI3K = phosphoinositide 3-kinase, AKT = protein kinase B, m-TOR = mechanistic Target of Rapamycin, MKK = mitogen-activated protein kinases, ERK = extracellular signal regulated kinase, JNK = c-Jun N-terminal kinase, BAX/BCL-2/BCL-X = apoptotic genes, ROS = reactive oxygen species, ΔΨm = mitochondrial membrane potential, HSP = heat shock protein, HO-1 = heme-oxygenase-1, Nrf2 = Nuclear factor (erythroid-derived 2)-like 2, STAT = signal transducer and activator of transcription, HIF-1α = hypoxia inducible factor 1α, NF-κB = nuclear factor κB.

A summary of the receptors and various proteins involved in argon’s molecular pathway may be found in Figure 1.

With an ongoing debate about the effects of lab-tested neuroprotective substances and their possible effects in clinically relevant settings, the time has come to get clinical trials started. Argon, as a non-toxic, easy-to-apply, odorless, available and cheap substance, with no reactive (chemical) binding partners in the common atmosphere, is one of the most promising volatile molecules to be tested in the near future. While it may be possible that cell- or animal-based results concerning neuroprotection in standardized models of injury are not fully convertible to a multifactorial setting of (mostly elderly) patients suffering from neuronal injury such as stroke and many other co-morbidities (including poly-medication, which may reduce the effect of any drug applied), it is most tempting to speculate that an inhalation of argon as fast as possible after onset of a stroke may alter neuronal apoptosis significantly, thus improving clinical outcome. This would not only reduce patient suffering from emotionally difficult rehabilitation therapy but also the economic burden on society. Any neuron to be rescued from apoptosis will potentially increase the chance of a restitutio ad integrum. Limitations of the proposed therapy with inhalation of argon include the uncertainty of the correct dose and the timing as well as the question of the duration of this therapy. Also, non-toxic, special inhalation systems should be provided to ensure that argon is inhaled by the patient at the specific dose without being affected by the surroundings.

## Figures and Tables

**Table 1:** Experimental analysis of the molecular mechanism of argon-mediated neuroprotection.

Ref.	Model/Setup	Cell Culture Animals	Groups (*n* = x)	Concentration Duration	Primary Outcome Parameter	General Results	Proposed Mechanism
Abraini 2003 [35]	Ar anesthesia under hyperbaric conditions	Adult Sprague Dawley rats	*n* = 6 *n* = 4	unknown	Loss of righting reflexes	gabazine and flumazenil reduced anesthetic action of Ar	GABA_A_ receptor
Harris 2013 [36]	1. Measurement of receptor currents 2. TBI to hippocampal brain slices	(a) HEK-293 cells (b) six day old C57/BL6 pups	*n* = 10 (NMDA) *n* = 5 (TREK) *n* = 105 (SHAM) *n* = 141 (TBI) *n* = 44 (Ar) *n* = 37 (Glycin + Ar)	Ar 80 Vol % 0.5 atm for 30 min up to 24 h	Currents via NMDA or TREK-1 receptor Quantification of cell inury	Ar does not affect NMDA or TREK currents Ar provides neuroprotection Pretreatment with Glycin did not alter Ar effects	NMDA receptor TREK-1 receptor
Brücken 2014 [37]	Cardiac arrest model	Adult Sprague Dawley rats	*n* = 47	Ar 40 Vol % and Ar 70 Vol %, each for 1 h	NDS, reduction of neuronal damage	Improvement of NDS after Ar inhalation K_ATP_ channel blocking had no effect	K_ATP_ channel
Fahlenkamp 2012 [41]	Ar with or without LPS exposure	BV-2 microglia primary neurons primary astrocytes	*n* = 3	Ar 50 Vol % 15, 30, 60 and 120 min	ERK1/2 phosphorylation cytokine expression	Ar activates ERK No effect on LPS mediated cytokine expression (IL-1β, TNF-α, IL-6)	ERK1/2 phosphorylation
Zhuang 2012 [25]	Hypoxic and ischemic brain injury	Sprague Dawley rat pups	5 groups *n* = 5–7	Ar 70 Vol % for 90 min, administration 2 h after injury	Infarct size and neurological function	Ar reduced infarction size and improved neurological function Ar increased BCL-2 expression	n.n.
Fahlenkamp 2014 [24]	tMCAO	Adult Sprague Dawley rats	4 groups *n* = 12–15	Ar 50 Vol % for 60 min administration 2 h after injury	Expression of growth factors and inflammatory cytokines	Ar increased: TGF-β, NGF, IL-6 and iNOS No effect on: HIF-1α, MMP-9, CNP, GFAP, VEGF-α, IGF-1 and microglia	n.n.
Zhao 2016 [47]	OGD Right common artery ligation and 90 min hypoxia	Cortical neuronal cell culture (SDR) Neonatal Sprague Dawley rats	*n* = 8	Ar 70/75 Vol % 2 h after hypothermia	Neuronal injury	Ar after hypothermia increased p-AKT, HO-1 and decreased Cytochrome C, Caspase-3, NF-κB and infarct size	n.n.
Zhao 2016 [50]	OGD Hypoxic and ischemic brain injury	Cortical neuronal cells of rat foetuses Sprague Dawley rat pups	5 groups *n* = 8	Ar 70 Vol % for 2 h after injury	Infarct size and protein expression	Ar reduced infarction size Different protein expression and inhibition of effects via U0126 (ERK inhibition)	Ar dependent activation of MAPK, p-mTOR, Nrf-2 and NQO1/SOD1
Höllig 2016 [28]	tMCAO	Adult Sprague Dawley rats	9 groups *n* = 9–11	Ar 50 Vol % for 60 min one hour after SAH	Mortality after SAH, neurological testing, protein analysis, quantification of neurons	Ar increased: HO-1 and HIF-1α expression No difference in neuroscore due to treatment	Ar dependent HO-1 and HIF-1α regulation
Ulbrich 2015 [45]	Retinal IRI	Adult Sprague Dawley rats	6 groups *n* = 8	Ar 75 Vol % for 60 min either immediately after IRI or with a 1.5 or 3 h delay	Vital retinal ganglion cells	Ar reduced HSP-70, HSP-90 and HO-1 expression, while inducing p38 and ERK1/2 ERK inhibition abolished Ar effects	ERK1/2
Ulbrich 2015 [38]	Rotenone induced apoptosis	SY5Y neuroblastoma cell line	*n* = 6	Ar 25/50 and 75 Vol % for 2 or 4 h after rotenone induced apoptosis	Reduction of apoptosis	Ar inhibited TLR2 and TLR4 receptors and downstream signaling in vitro	TLR2 and TLR4 signaling via IRAK4 and ERK1/2
Ulbrich 2016 [39]	Retinal IRI	SY5Y neuroblastoma cell line Adult Sprague Dawley rats	*n* = 6 6 groups *n* = 8	Ar 75 Vol % for 2 h Ar 75 Vol % for 60 min after IRI	Transcription factor analysis, Cytokine expression TLR2/TLR4 signaling	Ar inhibited STAT3 and NF-κB, but not STAT5 Ar affects mitochondrial membrane potential	TLR2 and TLR4 signaling in vivo via STAT3 and NF-κB pathway, suppressing IL-8

Abbreviations: Ar = argon, TBI = traumatic brain injury, min = minutes, h = hour(s), NDS = neurological dynsfunction score, n.n. = not named; LPS = lipopolysaccharide, tMCAO = transient mid cerebral artery occlusion, IRI = ischemia and reperfusion injury, MAPK = mitogen activated protein kinases, SAH = subarachnoid hemorrhage, OGD = oxygen-glucose deprivation, Vol % = percentage of volume, TNF-α = tumor necrosis factor-α, GABA_A_ = gamma aminobutyric acid A receptor, NMDA = *N*-methyl d-aspartate receptor, TREK-1 = potassium channel subfamily K member 1, IL-x = interleukin-x, NGF = nerve-growth factor, HIF-1α = hypoxia inducible factor 1α, MMP9 = matrix metalloproteinase-9, CNP = C-type natriuretic peptide, GFAP = glial fibrillary acidic protein, VEGF-α = vascular endothelial growth factor-α, AKT = protein kinase B, HSP = heat shock protein, HO-1 = heme-oxygenase-1, m-TOR = mechanistic Target of Rapamycin, NQO1 = NADPH quinone dehydrogenase-1, SOD1 = superoxide dismutase 1, ERK = extracellular signal-regulated kinase, TLR = toll-like receptor, IRAK = interleukin-1 receptor-associated kinase, Nrf2 = Nuclear factor (erythroid-derived 2)-like 2, STAT = signal transducer and activator of transcription, NF-κB = nuclear factor κB.
